# Prediction of Ammonia Concentration in a Pig House Based on Machine Learning Models and Environmental Parameters

**DOI:** 10.3390/ani13010165

**Published:** 2022-12-31

**Authors:** Siyi Peng, Jiaming Zhu, Zuohua Liu, Bin Hu, Miao Wang, Shihua Pu

**Affiliations:** 1 Chongqing Academy of Animal Sciences, Changlong Avenue, Chongqing 402460, China; 2 College of Animal Science and Technology, Southwest University, Chongqing 402460, China; 3 National Center of Technology Innovation for Pigs, Chongqing 402460, China; 4 Scientific Observation and Experiment Station of Livestock Equipment Engineering in Southwest, Ministry of Agriculture and Rural Affairs, Chongqing 402460, China; 5 Innovation and Entrepreneurship Team for Livestock Environment Control and Equipment R&D, Chongqing 402460, China

**Keywords:** ammonia concentration, machine learning, prediction models, pig house

## Abstract

**Simple Summary:**

With the increased development of pig farming intensification, air quality and odor emissions in pig houses are gradually attracting attention. Among them, ammonia is considered to be an important environmental indicator of pig house. Excessive accumulation of ammonia can seriously affect the growth status of pigs and also cause a potential health risk to farm workers. Therefore, it is very important to recognize the changes of ammonia in pig houses and to discharge ammonia in time for the welfare farming of pigs. In this study, three traditional machine learning algorithms and three deep learning algorithms were selected to predict the ammonia concentration in a pig house. Based on them, important environmental parameters and promising algorithms were screened out and the algorithms were evaluated for optimization. The results of the study can provide a reference for air quality regulation in pig houses.

**Abstract:**

Accurately predicting the air quality in a piggery and taking control measures in advance are important issues for pig farm production and local environmental management. In this experiment, the NH_3_ concentration in a semi-automatic piggery was studied. First, the random forest algorithm (RF) and Pearson correlation analysis were combined to analyze the environmental parameters, and nine input schemes for the model feature parameters were identified. Three kinds of deep learning and three kinds of conventional machine learning algorithms were applied to the prediction of NH_3_ in the piggery. Through comparative experiments, appropriate environmental parameters (CO_2_, H_2_O, P, and outdoor temperature) and superior algorithms (LSTM and RNN) were selected. On this basis, the PSO algorithm was used to optimize the hyperparameters of the algorithms, and their prediction performance was also evaluated. The results showed that the *R^2^* values of PSO-LSTM and PSO-RNN were 0.9487 and 0.9458, respectively. These models had good accuracy when predicting NH_3_ concentration in the piggery 0.5 h, 1 h, 1.5 h, and 2 h in advance. This study can provide a reference for the prediction of air concentrations in pig house environments.

## 1. Introduction

In intensive and large-scale pig production, air quality and odor emissions have a negative impact on the health of the pigs, the pig farm workers, and the local environment. Ammonia (NH_3_) concentration is an important indicator used to evaluate the environment of a piggery. The high concentration of NH_3_ in the piggery will affect the normal growth of pigs, resulting in decreased immunity and production performance and inducing respiratory diseases [1]. Excretion of NH_3_ from pig houses may pose a risk of respiratory illness to pig farm workers and residents living nearby [2]. When NH_3_ is excessively discharged into the atmosphere, it returns to the surface through atmospheric dry and wet deposition processes, causing acidification of soil and water bodies and affecting ecosystem stability [3]. Therefore, the development of tools to assist managers in anticipating changes in NH_3_ concentration in a piggery will ensure that timely measures can be taken to reduce the potential stress of ammonia on human and animal health, and the level of environmental pollution, factors that are important to improve animal production, animal welfare, and environmental management.

In the past, statistical models such as least squares extensive and stepwise linear regression were developed for gas concentration prediction in aquaculture environments [4,5]. However, air pollutants in farms are mixed, complex, and usually have interaction characteristics that lead to the concentrations of air pollutants having non-linear dynamics [6]. Therefore, many statistical models in the past have poor prediction of gas pollution concentration in farms. Machine learning (ML) algorithms can deal with nonlinear interactions mathematically, and they have excellent performance in feature extraction, classification, and change prediction for big data. Machine learning has been developed rapidly in recent years [7,8,9]. Classical machine learning algorithms include neural networks and decision trees (DT). Based on these models, random forest (RF), extreme gradient boosting (XGBoost), backpropagation neural networks (BPNN), Elman neural networks (RNN), long short-term memory (LSTM), and other algorithms have been developed [10,11]. These algorithms have been applied to the prediction and regulation of environmental factors such as the automation of indoor air management, greenhouse gas emissions, and air pollution assessment, and have achieved good results [12,13].

Although a few researchers have constructed air prediction models for farming environments based on machine learning algorithms in recent years, the environments in farming houses vary greatly from region to region, and numerous modeling attempts and screenings are needed to achieve extensive gas concentration prediction [14,15]. For example, many pig houses have started to adopt the regulation mode (called “semi-automatic regulation” in this paper) that automatically changes the ventilation rate based on the set house temperature value. In this mode, the temperature fluctuation in the house is low, but the concentration of air pollutants in the house is often still too high in autumn and winter, and there are very few corresponding models for predicting air pollutants. In addition, as far as the modeling process is concerned, the selection of machine learning algorithms and environmental parameters in feature engineering are key aspects in determining the performance of the model, and there are very few relevant reports concerning the farming environment that can draw on how to select the underlying algorithms and environmental parameters.

In this study, we evaluated the ability of three traditional machine learning and three deep learning algorithms to predict NH_3_ concentration in a semi-automatically regulated pig house in combination with environmental parameters. The traditional machine learning algorithms include the classical DT, as well as support vector machine (SVM) and XGBoost, which have performed well in the past for gas prediction in farming environments [16,17]. Deep learning algorithms were chosen from the common BPNN, as well as LSTM and RNN, as these models have strong regression capabilities for time series data but are rarely employed in farming environments [18,19]. For the selection of environmental parameters, the three most concerned parameters (indoor temperature, humidity, and ventilation) in the pig house were measured, as well as the temperature and rainfall outside the house, as the latter can well reflect the changing state of the natural environment outside the house. In addition, from the response principle, indoor air pressure (P), H_2_O, and CO_2_ may also have an effect on NH_3_ concentration, and these three indicators were also included in the monitoring of environmental parameters [20]. The main objectives were to evaluate the performance of LSTM, RNN, BPNN, DT, SVM, and XGBoost in predicting NH_3_ concentrations in semi-automatically regulated pig houses, and to identify the main environmental factors affecting NH_3_ concentration. On this basis, two models with strong performance in predicting NH_3_ concentration in semi-automatic pig houses were proposed and optimized. This study can be a reference for future work related to gas concentration prediction in different farming modes.

## 2. Materials and Methods

### 2.1. Data Collection

This study was conducted in a fattening pig house of a pig farm in Rongchang, Chongqing. More detailed information concerning this house is given in Pu et al. [21].

Environmental data were collected from 17 September 2020, to 20 October 2020. During this period, a total of 220 pigs in the pig house were evenly distributed in 22 pens, with each pig weighing 70–90 kg. An INNOVA (model 1412I, LumaSense, Inc., USA) based on the detection principle of infrared photoacoustic spectroscopy was used to monitor and record the data of NH_3_, CO_2_, and H_2_O every 3 min. The HOBO (U23-001, Onset, Bourne, MA, USA) was used to monitor temperature and relative humidity, and was set to record every 5 min. The monitoring points of the above indexes were near 1.7 m in the middle of the pig house channel. Meanwhile, the ventilation volume in the piggery was regulated and recorded automatically by the intelligent system (Chongqing Dahong Machinery Co., Ltd., Chongqing, China) inside the piggery. Moreover, the temperature and rainfall data outside the house were recorded by surrounding small meteorological stations.

### 2.2. Data Preprocessing

In order to ensure the prediction performance of the model, the data collected by the equipment inside and outside the piggery and the intelligent system inside the piggery were preprocessed and analyzed. First, abnormal data processing was carried out on the environmental parameter data of the pig house using Formula (1). If the absolute value of the difference between the value and its average value was greater than three times its standard deviation, the value was replaced by the average value of the data on both sides of the value. Then, the environmental parameter data were averaged for half an hour using Formula (2). Because the dimensions of sampling equipment in the piggery were different, Equation (3) was used to normalize the data.

(1) $$\left|y_{n}-y^{\prime }\right|>3\sigma \;\;\;\;\;y_{n=}\frac{y_{n-1}-y_{n+1}}{2},$$

(2) $$y_{h}=\frac{\left(y_{1}+y_{2}+\ldots +y_{n}\right)}{\left(30/t\right)},$$

(3) $$y^{*}=\frac{\left(y_{n}-y_{min}\right)}{\left(y_{max}-y_{min}\right)}.$$

Here, $y_{n}$ is the collected value of a pig house sensor; $y^{\prime }$ is the mean value of the sensor data sequence; $y_{n}$ is the data value after abnormal data processing; σ is the standard deviation of sensor data sequence; *n* is the data point; $y_{h}$ is the value after averaging every 30 min; *t* is the sensor acquisition time interval; $y_{max}$ is the maximum value of the sensor data sequence; $y_{min}$ is the minimum value of the sensor data sequence, and $y^{*}$ is the normalized value.

### 2.3. Model Construction

**Figure 1:**
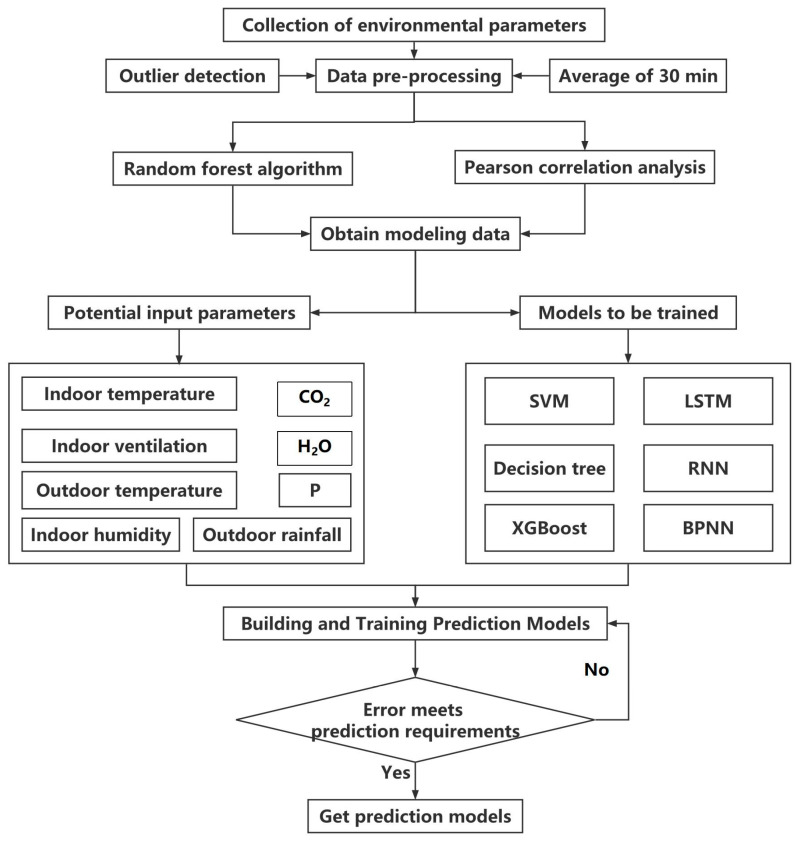
Modeling workflow.

The construction process of the six prediction models was consistent (Figure 1), and they were all carried out in the following three steps: selecting the environmental parameters to determine the feature input scheme (2.3.1), selecting and importing potential algorithms from scikit-learn or Keras libraries using Python (2.3.2), and training the input data based on different algorithms and adjusting parameters in combination with model evaluation metrics to achieve relatively good results (2.3.3).

#### 2.3.1. Selection of Input Environmental Parameters

A variety of environmental parameters concerning the piggery were collected to build the model, including temperature, humidity, CO_2_, H_2_O, ventilation, air pressure inside the pig house, and temperature and rainfall outside the pig house. These eight parameters were considered potentially correlated variables. On this basis, the random forest algorithm was used to rank the importance of eight environmental parameters on NH_3_ concentration in the pig house. Random forest can yield the importance score of each variable to evaluate the role of each in classification, as it relies on a self-help resampling technology and node random splitting. The ability to analyze complex interacting classification features makes random forest a feature selection tool for high-dimensional data. In this study, we considered the parameters with importance scores greater than 0.1 after random forest analysis as the priority input environmental parameters, and selected the inputs in order of importance from the largest to the smallest. The environmental parameters with importance scores less than 0.1 were used to calculate their correlations with NH_3_ concentration using Pearson correlation analysis (PsCA), and the inputs were selected in order from the largest to the smallest according to the absolute value of correlation. The input scheme for the model characteristic parameters was obtained on the basis of the analysis of environmental importance and the correlations among the data (Table 1).

#### 2.3.2. Model Selection and Import

The NH_3_ concentration of the pig house was used as the label datum, and the environmental parameters related to the NH_3_ concentration were used as the characteristic data. The purpose was to learn the correspondence from the characteristic data such as temperature and humidity to predict the label data. Therefore, it was necessary to model the supervised learning algorithm in machine learning. At the same time, the input variables and output variables were time series, so the prediction of NH_3_ in the pig house was formally a regression problem, and the corresponding model is a non-probabilistic model. Therefore, different machine learning algorithms were used to establish discriminant models in supervised learning, including classical algorithms such as neural networks, DT, SVM, and related ensemble algorithms (XGBoost, LSTM, RNN, BPNN). Using Python software, machine learning algorithm running, statistical analysis, and data mining work were managed with pandas, matplotlib, and numpy. Traditional machine learning algorithms (DT, SVM, and XGBoost) were imported directly from the scikit-learn library and combined with the input data for subsequent training and hyperparameter optimization, while deep learning algorithms (BPNN, LSTM and RNN) required additional use of the Keras library and artificial debugging to determine the number of hidden layers (there were two hidden layers in this study).

#### 2.3.3. Model Training

The NH_3_ concentration was used as the prediction target. The length of the input time series (input_len) of each model was set to 5, and the length of the prediction time series (out_len) was set to 1. The first 80% of the preprocessed data was used to train the model, and the last 20% was used to test the model. In the training process, the training of each integrated model involved the selection of hyperparameters, a factor that is directly related to the final prediction results. Here, the hyperparameters were firstly artificially selected and set so that the prediction effect was relatively high, and then three deep learning models and three conventional machine learning models were established. Then, the models with good prediction performance were screened, and hyperparameter optimization was performed using the corresponding algorithms on this basis. For neural network algorithms (LSTM, RNN, and BPNN), the particle swarm optimization (PSO) algorithm was used to optimize the number of hidden layer neurons in the first and second layers and the learning rate. For DT, SVM, and XGBoost algorithms, grid search was used for parameter tuning. 

### 2.4. Model Performance Evaluation

The performance of the models was evaluated with mean absolute error (MAE), root-mean-squared error (RMSE), and coefficient of determination ($R^{2}$), which are shown in Equations (4) and (5), respectively.

1. Root-Mean-Squared Error (RMSE)

(4) $$RMSE=\sqrt{\frac{1}{n}\overset{n}{\underset{i=1}{\sum }}{\left(y_{oi}-y_{pi}\right)}^{2}}.$$

As with MAE, a smaller RMSE means that the model prediction performance is better.

2. Coefficient of Determination ($R^{2}$)

(5) $$R^{2}=\frac{\sum_{i=1}^{n}{\left(y_{pi}-y_{om}\right)}^{2}}{\sum_{i=1}^{n}{\left(y_{pi}-y_{om}\right)}^{2}+\sum_{i=1}^{n}{\left(y_{oi}-y_{pi}\right)}^{2}},$$

where *y_om_* is the mean value of the observed value. An $R^{2}$ closer to 1 means the model is better.

## 3. Results and Discussion

### 3.1. Data Characteristics

**Figure 2:**
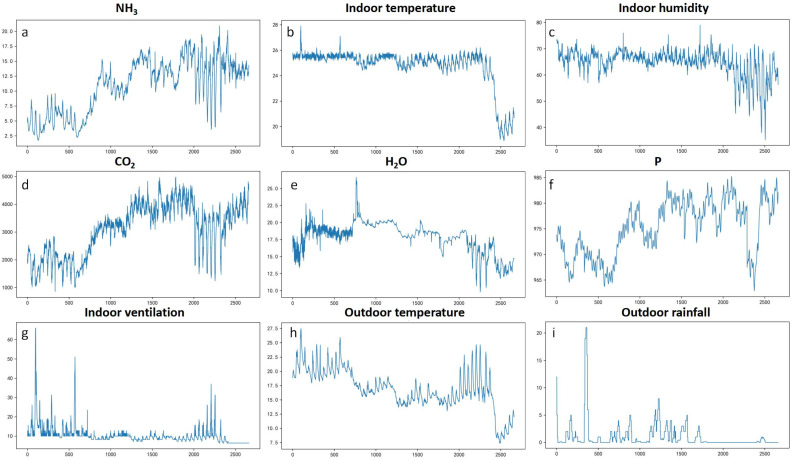
Internal and external environment of the pig house. The above graphs show the concentration of NH_3_ (**a**), CO_2_ (**d**) and H_2_O (**e**) inside the house, as well as the temperature (**b**), humidity (**c**), air pressure and ventilation (**f**,**g**) inside the house, and the temperature and rainfall (**h**,**i**) outside the house, respectively.

The collected parameter information is shown in Figure 2. The average concentration of NH_3_ fluctuated in the range of 1.77–20.94 ppm, and its association with CO_2_ concentration showed a significant upward trend from the 550th to the 2000th time point. Interestingly, the outdoor temperature and ventilation rate were opposite to the change trends of NH_3_ and CO_2_ concentration, fluctuating in the range of 7.5–27.0 °C and 6.0–67.5 m^3^/min, respectively. The temperature and humidity in the house were relatively stable in the first 2000 time points, fluctuating in the range of 23.5–28.0 °C and 57.6%–78.5%, respectively. From the 2000th to the 2300th time point, humidity in the house fluctuated significantly, and the environmental parameters near the time period changed as well, including a short-term rise in the temperature outside the house, a short-term increase in the ventilation volume in the house, and fluctuation of humidity, air pressure, NH_3_ concentration, and CO_2_ concentration in the house.

The concentration of NH_3_ met the standards of 25 mg·m^−3^, while the CO_2_ did not meet the respective standard of 1500 mg·m^−3^ as prescribed by The Ministry of Agriculture of the People’s Republic of China, given in NY/T 17824.3-2008 “Environmental parameters and environmental management for intensive pig farms.” The semi-automatic control of the piggery in this experiment was able to automatically control the ventilation rate based on the temperature, so the temperature in the piggery remained relatively stable for most of the time. At the same time, when the temperature outside the house decreases, the ventilation inside the house is subsequently reduced, which in turn allows air pollutants to start accumulating in the pig house [22,23]. This is perhaps the main reason why NH_3_ and CO_2_ concentrations gradually increased after the 550th time point. It is worth noting that there was a brief increase in the outside temperature from the 2000th to 2300th time points, and the ventilation rate of the house increased automatically; this may also be the reason for the decreases in NH_3_ and CO_2_ concentrations at this time point. Thus, it seems that excessive concentrations of air pollutants in the pig house can occur, and a timely increase in ventilation in the pig house can effectively control the environment to a certain extent.

### 3.2. Importance and Correlation of Environmental Parameters

**Figure 3:**
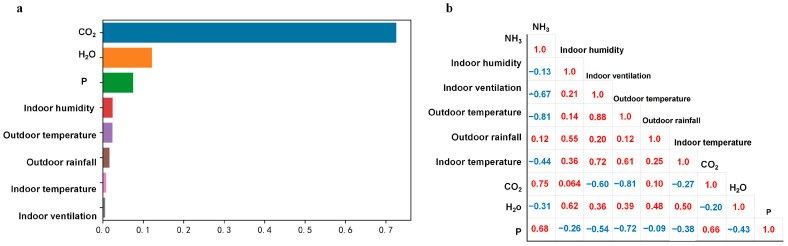
Analysis of piggery environment variables based on random forest (**a**) and Pearson correlation (**b**).

The RF algorithm was used to evaluate the environmental variables affecting the concentrations of air pollutants in the piggery, and the importance of each variable was obtained and sorted (Figure 3a). The most important influence on NH_3_ concentration was CO_2_ concentration (importance of 0.73), followed by H_2_O and P (0.12 and 0.07, respectively). Humidity, outdoor rainfall, temperature, and indoor ventilation were less important. Considering that the RF algorithm may be omitted in parameter screening, a PsCA was performed between the concentrations of gaseous pollutants in the piggery and various environmental variables (Figure 3b). The results showed that there was a strong positive correlation between CO_2_ and NH_3_ concentrations (+0.75), followed by a strong positive correlation between P and NH_3_ concentrations (+0.68). At the same time, there was a strong negative correlation between outdoor temperature and NH_3_ concentration (−0.81), and there were also strong negative correlations between indoor ventilation and temperature and NH_3_ concentration (−0.67 and −0.44, respectively).

The importance of CO_2_ to NH_3_ concentration may be due to the formation of CO_2_ during NH_3_ production. Uric acid decomposition is the main source of NH_3_ in a piggery [24]. Uric acid is hydrolyzed into urea and glyoxylic acid under the action of various microorganisms, and finally urea produces NH_3_ and CO_2_ under the action of urease [20]. In addition, NH_3_ emissions need to be transmitted through the liquid film layer of the air to the gas film layer, and finally enter the external atmospheric environment. This process will be accompanied by H_2_O volatilization, and this may be the main reason why H_2_O had an impact on the NH_3_ concentration in the pig house. Random forest is a classifier established in a random manner and contains multiple decision trees [25]. Although the algorithm has been verified to effectively evaluate the contribution of environmental parameters to the indicators, there may be multiple similar decision trees in the piggery environment [26,27]. If there are several environmental parameters that are important for NH_3_ concentration because of the same mechanism, then some of them are likely to be neglected in the random forest method. Therefore, we introduced PsCA and found that P, indoor temperature, outdoor temperature, and indoor ventilation had high correlations. P changes with the external atmospheric environment and the ventilation volume in the piggery, and this may be the reason P had strong positive correlations with the outside temperature and the ventilation in the piggery. In addition, ventilation rate is an important parameter for regulating the environment of the piggery. In this study, the ventilation rate was set to increase or decrease according to the temperature inside the piggery, and the temperature inside the piggery would change with the infiltration of the temperature outside the piggery; this may be the reason for the large negative correlations between the temperature outside the piggery, the temperature inside the piggery, the ventilation rate, and NH_3_ concentration. In general, there were interactions among environmental parameters in the pig house.

### 3.3. Model Comparison

**Figure 4:**
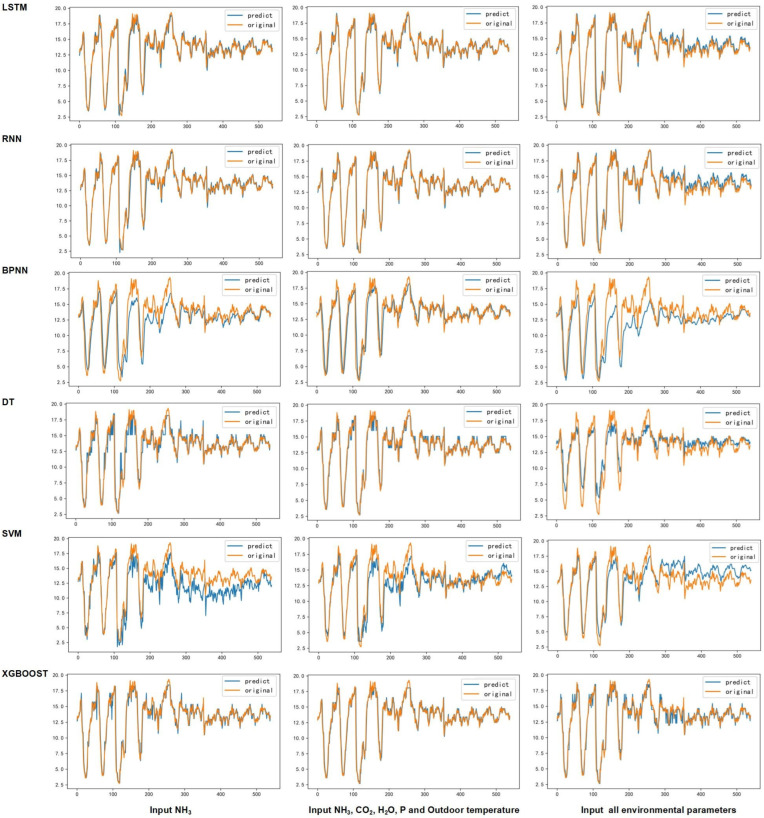
Comparison between predicted and original values of each model after the input of only NH_3_ (first column), input of NH_3_, CO_2_, H_2_O, P, and Outdoor temperature (second column) and input of all environmental variables (third column).

According to the analysis performed for the environmental parameters, nine input schemes of characteristic parameters were determined in the process of training the model (Table 1), and the accuracy of each model was evaluated with the value of *R^2^* as the index (Table 2). Meanwhile, three cases were selected for comparative analysis without feature parameters (only input NH_3_), partial characteristic parameters with good prediction effect (input NH_3_, CO_2_, H_2_O, P, and outdoor temperature), and full characteristic parameters were selected for comparative analysis (Figure 4). In general, LSTM, RNN, and XGBoost had excellent prediction results, and even with different input features; the predicted and original values of these three models in the test set mostly overlapped, especially LSTM and RNN (Table 2 and Figure 4). DT could partially predict NH_3_ concentration, but the difference between its predicted and original values was larger than those of the first three. BPNN had good prediction results only when suitable input features (such as input NH_3_, CO_2_, H_2_O, P, and outdoor temperature) were used, and it deviated from the overall performance of both SVM. When the input feature was only NH_3_, the LSTM, RNN, and XGBoost could mostly predict NH_3_ (the first column in Figure 4), and most of their predictions differ from the original values only at the inflection point. When the input features were NH_3_, CO_2_, H_2_O, P, and outdoor temperature, the LSTM, RNN, and XGBoost models produced better prediction results than others. The predicted values of the six models were closer to the original values (the second column of Figure 4) than when only NH_3_ was input. The predicted values of LSTM, RNN, and XGBoost coincided with the original values at most of the inflection points. When all environmental parameters were used as input features (the third column of Figure 4), even for the LSTM and RNN, the deviation of the predicted values from the original values increased at the 300th time point of the test set. The difference between predicted and original values increased for the six models compared to when only NH_3_ was input.

LSTM and RNN have been considered as powerful algorithms for predicting atmospheric pollutant concentrations in previous studies [28,29]. XGBoost is a typical tree model for unstable classifiers that can solve nonlinear problems and has achieved good results in indoor odor prediction in the past [30,31]. In this study, when the input environmental parameters were the same, all three of the above algorithms showed strong predictive power in most cases, especially LSTM and RNN. The RNN algorithm is a kind of feedforward neural network that can transmit signals from input to output in only one way, and it introduces the self-connections of a neural cyclic structure into the network [32,33]. Therefore, the algorithm has good predictive power for data with serial characteristics. LSTM is based on RNN by introducing memory blocks to overcome vanishing and exploding gradients [34]. The memory block consists of three gating units: an input gate, an output gate, and a forget gate, where the input gate controls the flow of cell activation from the input to the memory cell, and the output gate controls the flow of output from the memory cell to other nodes [35]. Considering that both LSTM and RNN performed better than other models in this study for the nine input schemes, the results suggest that both LSTM and RNN models may have good prediction ability for NH_3_ concentration in semi-automated pig houses.

When the input environmental parameters were altered, the *R^2^* values of the models, even those constructed using the same algorithm, could be dramatically different. In this study, the *R^2^* of each model with input NH_3_, CO_2_, H_2_O, P, and outdoor temperature were improved compared to when only NH_3_ was input, especially for BPNN and SVM. This is consistent with previous studies that environmental parameters could increase model accuracy [36,37]. It is noteworthy that the *R^2^* value of each model decreased when all environmental parameters were input than when only NH_3_ was input. This could be that some of the features were not strongly correlated with changes in NH_3_ concentration and instead negatively affected the models when they were trained [38]. In general, the input of some environmental feature parameters can improve the model accuracy, although the number of feature parameters input needs to be controlled, and suitable indicators need to be selected. For the prediction of NH_3_ concentration in semi-automated pig houses, the characteristic parameters may firstly be considered as indicators with high importance after random forest analysis, and secondly be considered as supplementary from the perspective of correlations.

### 3.4. Model Optimization and Evaluation

Based on the analysis results of Section 3.3, LSTM and RNN models were further optimized. Here, both models comprised two hidden layers, and the number of neurons in the first and second hidden layers and the learning rate were determined by the PSO algorithm. The hyperparameters and evaluation indexes after model optimization are shown in Table 3. After optimization by PSO algorithm, both LSTM and RNN models were improved. The *R^2^* values of PSO-LSTM and PSO-RNN increased to 0.9487 and 0.9458, respectively. In addition, LSTM and RNN were tried in combination (PSO-LSTM-RNN). The weights of the PSO-LSTM-RNN model were obtained by the optimal weighting method, and the final prediction value of the ammonia concentration in the piggery was obtained. The prediction error of the PSO-LSTM-RNN model was very close to that of PSO-LSTM and PSO-RNN, and the *R^2^* and *RMSE* values of this model were 0.9416 and 0.5893, respectively.

To further evaluate the predictive power of the optimized model, the PSO-LSTM, PSO-RNN and PSO-LSTM-RNN models were applied to the prediction at different time scales. The input length of each model was set to 15, and the output lengths were set to 1, 2, 3, 4, 5, and 6; in other words, the prediction of NH_3_ concentration in the piggery after 0.5 h, 1 h, 1.5 h, 2 h, 2.5 h, and 3 h were realized. As seen in Table 4, all three models showed strong prediction ability for ammonia concentrations in pig houses in the next 2 h (corresponding to the next 1–4 time points), especially for 0.5 h, 1 h, and 1.5 h (*R^2^* values > 0.93; *RMSE* < 0.91). With the increase in prediction time, the predicted value deviated from the actual value, and the overall prediction error became greater. When predicting the NH_3_ concentration of the piggery after 2.5 h (corresponding to more than five time points), The *R^2^* values for the PSO-LSTM, PSO-RNN, and PSO-LSTM-RNN models decreased below 0.9, while *RMSE* increased for each model.

The PSO algorithm, which originated from the study of social behavior of birds and fish, is an intelligent evolutionary computational method that relies on collaboration and information sharing among individuals in a population to find the optimal solution [39]. In this algorithm, each particle is a moving individual in the N-dimensional search space, and the particle has two attributes: velocity and position. A particle adjusts its position in the search space and collaborates with other particles to calculate the global optimal solution. The PSO algorithm has been widely used in the field of machine learning algorithms because of its computational simplicity and high convergence efficiency [40]. In this study, the PSO algorithm was applied to the optimization of LSTM, RNN, and LSTM-RNN, and the relatively good values for the hyperparameters of the two models were determined. The algorithm effectively improved the model accuracy.

All three models optimized by the PSO algorithm showed high accuracy (*R^2^* > 0.9) in predicting 1–4 future time points; this result may indicate that these types of models have good prospects in application to the prediction of NH_3_ concentration in pig houses at different time scales. At the same time, the accuracy of all three models was very close at all time scales, possibly due to the similarity of the LSTM and RNN algorithms [41]. However, the advantage of the higher accuracy of LSTM on long time series data was not found in this study. This may have been due to the fact that the NH_3_ concentration in this experiment was influenced by artificial regulation from time to time, and this in turn made the pattern of NH_3_ changes over longer times behave unpredictably. In addition, for PSO-LSTM-RNN, although the number of hidden layers was increased to three during the construction of this model, a setting that was somewhat different from the single model, the accuracy of the model was not significantly improved after the combination of the two; however, this modification did increase the complexity of the model and the computer operation burden, which are factors that related to the similarity of the principles of RNN and LSTM algorithms. Overall, the PSO-LSTM and PSO-RNN models could effectively predict NH_3_ concentrations in a pig house at four future time points with a balance of model complexity and accuracy, and thus they have good application prospects. In addition, if a further combination of models is needed in the future, it may be necessary to consider model construction from the perspective of synergy or complementarity between algorithms.

## 4. Conclusions

There are complex interactions among various environmental parameters in a piggery. In this study, random forest and PsCA were used to retain important characteristic parameters as much as possible while controlling the input variables of the model. Through comparative experiments, it was found that after inputting appropriate environmental parameters (e.g., CO2, H2O, P, and outdoor temperature) the accuracy of each model for predicting ammonia concentrations was superior to that when only NH_3_ was input, while the accuracy of each model decreased after inputting too many environmental parameters. The LSTM and RNN models were selected, which were able to effectively predict the NH_3_ concentration in a semi-automatic pig house. On this basis, the PSO-LSTM and PSO-RNN models were proposed by using the PSO algorithm. These models were more accurate than LSTM and RNN and had a good prediction effect on NH_3_ concentration at different time scales. The PSO-LSTM and PSO-RNN models have excellent potential for application in predicting gas concentrations in breeding environments, and the introduction of other algorithms in terms of complementarity or synergy can be considered candidates with which to build more powerful combined models.

## Figures and Tables

**Table 1:** Input scheme of model feature parameters.

Serial Number	Input Parameters
1	NH_3_
2	NH_3_, CO_2_
3	NH_3_, CO_2_, H_2_O
4	NH_3_, CO_2_, H_2_O, P
5	NH_3_, CO_2_, H_2_O, P, Outdoor temperature
6	NH_3_, CO_2_, H_2_O, P, Outdoor temperature, Indoor ventilation
7	NH_3_, CO_2_, H_2_O, P, Outdoor temperature, Indoor ventilation, Indoor temperature
8	NH_3_, CO_2_, H_2_O, P, Outdoor temperature, Indoor ventilation, Indoor temperature, Indoor humidity
9	NH_3_, CO_2_, H_2_O, P, Outdoor temperature, Indoor ventilation, Indoor temperature, Indoor humidity, Outdoor rainfall

**Table 2:** Evaluation of model accuracy via *R^2^* for each of the nine feature input schemes.

Input Feature Parameters	Model Algorithm					
	LSTM	RNN	BPNN	DT	SVM	XGBoost
Serial 1	0.9239	0.9176	0.5709	0.8973	0.7240	0.9171
Serial 2	0.9297	0.9214	0.8080	0.8993	0.8948	0.9234
Serial 3	0.9348	0.9327	0.7999	0.9060	0.8975	0.9267
Serial 4	0.9335	0.9275	0.5726	0.8977	0.8078	0.9173
Serial 5	0.9321	0.9392	0.8241	0.9067	0.9137	0.9312
Serial 6	0.9115	0.9138	0.5034	0.8949	0.6853	0.9077
Serial 7	0.9183	0.9197	0.6226	0.8872	0.7875	0.9095
Serial 8	0.8780	0.8739	0.4953	0.8652	0.7899	0.8707
Serial 9	0.9102	0.9007	0.4289	0.8683	0.7755	0.8861

**Table 3:** The values of hyperparameters and prediction errors of LSTM and RNN models after optimization by PSO algorithm.

Model	Hyperparameters			Prediction Errors	
	Dense1	Dense2	Learning Rate	*RMSE*	*R^2^*
PSO-LSTM	100	259	0.001	0.5914	0.9487
PSO-RNN	100	339	0.0007	0.6125	0.9458

**Table 4:** Prediction errors of PSO-LSTM and PSO-RNN models at different time scales.

Model	Time Scales	*RMSE*	*R^2^*
PSO-LSTM	0.5 h	0.8626	0.9447
	1 h	0.8328	0.9382
	1.5 h	0.9105	0.9378
	2 h	1.0297	0.9182
	2.5 h	1.1729	0.8968
	3 h	1.2264	0.8773
PSO-RNN	0.5 h	0.8273	0.9433
	1 h	0.8838	0.9417
	1.5 h	0.8703	0.9353
	2 h	1.0301	0.9169
	2.5 h	1.1256	0.8856
	3 h	1.2651	0.871
PSO-LSTM-RNN	0.5 h	0.8448	0.9441
	1 h	0.8583	0.9398
	1.5 h	0.8951	0.9361
	2 h	1.0296	0.9176
	2.5 h	1.1471	0.8912
	3 h	1.2458	0.8761

## Data Availability

The data that support the findings of this study are available from the corresponding author, upon reasonable request.
